# Autistic adults’ inclination to lie in everyday situations

**DOI:** 10.1177/13623613231183911

**Published:** 2023-08-12

**Authors:** Ralph Bagnall, Ailsa Russell, Mark Brosnan, Katie Maras

**Affiliations:** University of Bath, UK

**Keywords:** autism, deception, memory, social cognition and social behaviour, theory of mind

## Abstract

Autistic children and adolescents often have greater difficulty engaging in deception than their non-autistic peers. However, deception in autistic adulthood has received little attention to date. This study examined whether autistic and non-autistic adults differed in their inclination to lie in everyday situations and the factors that underpin this. Forty-one autistic and 41 non-autistic participants completed self-report measures relating to their inclination to lie, ability to lie and moral attitudes about the acceptability of lying. Participants also undertook a reaction-time test of lie-telling, as well as theory of mind and working memory measures. Autistic and non-autistic adults did not significantly differ in their inclination to lie in everyday situations. The degree to which lying was viewed as morally acceptable positively predicted both groups’ inclination to lie. The remaining factors underpinning the inclination to lie differed between groups. Lower self-rated lying ability and slower lie speed predicted a reduced inclination to lie in autistic participants, whereas higher theory of mind and working memory capacity predicted a reduced inclination to lie in the non-autistic group. Implications for our understanding of deception in autistic and non-autistic adults are discussed.

**Lay abstract:**

Differences in social communication and understanding others’ mental states may mean that autistic adults are less likely to deceive others than non-autistic individuals. We investigated whether autistic and non-autistic adults differ in their inclination to lie and which psychological factors are involved in the inclination to lie. We found that autistic and non-autistic groups reported a similar inclination to lie, and the extent to which participants viewed lying as acceptable helped to explain their inclination to deceive others. However, the other underlying psychological factors associated with deception inclination differed between autistic and non-autistic groups. Autistic adults’ belief about their ability to lie and also how quickly they could lie helped to explain whether they were more or less inclined to lie. For non-autistic adults, their memory and ability to understand others’ mental states helped to explain their lie inclination. We discuss these findings and recommend areas for future research.

Deceptive behaviour by autistic children and adolescents has long been of psychological interest. Over the past three decades, Theory of Mind (ToM) – the capacity to understand others’ mental states (Baron-Cohen et al., 1985; Premack & Woodruff, 1978) – and executive functioning – interrelated psychological processes involved in goal-orientated cognition (Diamond, 2013) – have each been proposed to explain the reported difficulties autistic people can have with lying (see Bagnall et al., 2022 for review). Beyond socio-cognitive models, some autistic adolescents also describe an inherent value of truthfulness over lying (Atherton et al., 2019). Two recent studies have empirically examined deception by autistic adults (without co-occurring intellectual disability), building upon previous research with children and adolescents. In the study by Bagnall et al. (2023), autistic adults displayed similar verbal deception behaviour (e.g. quantity of detail) as non-autistic adults when they were instructed to lie during a mock-police suspect interview. Furthermore, in the study by van Tiel et al. (2021), autistic adults were often more likely to successfully deceive a computerised opponent than non-autistic adults (i.e. demonstrating lying ability). Autistic adults were also equally likely to deceive a computerised opponent as non-autistic adults (i.e. showing inclination to lie). However, van Tiel et al. (2021) emphasised that the non-social (computerised) nature of the task may have aided autistic participants’ performance, and therefore may not translate to everyday, social settings.

Lab-based deception often differs from lying in everyday life, in which to lie is a decision influenced by communication goals and socio-ecological context (see Levine, 2018). According to Ryan et al. (2019), expectancy-value theories (Barron & Hulleman, 2015; Vroom, 1964; Wigfield & Eccles, 2000) propose that motivation for a given behaviour or task is influenced by an individual’s expectation of success, their perception of task value and the degree of effort required to succeed, such as cognitive load (see Feldon et al., 2019). Thus, despite findings from recent studies (Bagnall et al., 2023; van Tiel et al., 2021), autistic adults may be less inclined to lie in everyday situations due to factors not yet examined in previous literature. For example, autistic adults’ beliefs about their own deception ability (i.e. expectation of success), how morally acceptable they perceive lying to be (i.e. perception of value) and the degree to which lying is cognitively demanding (i.e. effort and/or cost) may all influence a broader inclination to lie. As interpersonal deception becomes increasingly socio-cognitively complex throughout childhood, adolescence and adulthood (Walczyk & Fargerson, 2019), it may therefore be assumed that autistic adults are broadly less inclined to lie in everyday situations than their neurotypical peers.

As outlined, self-related and moral beliefs may contribute to differences between autistic and non-autistic adults’ inclination to lie. Non-autistic adults who rate their own lie-telling ability more highly also report lying more frequently (Verigin et al., 2019). There is currently little known about autistic adults’ beliefs about their ability to lie, though some autistic adults have described lying as anxiety-inducing and physically discomforting (Jaarsma et al., 2012). Alternatively, autistic adults may consider themselves poorer liars due to a tendency to downplay social cognitive ability. For instance, DeBrabander et al. (2021) reported that autistic adults tend to perceive their own social cognitive task performance more poorly than how they expect the ‘average person’ will perform. This is despite autistic adults’ aforementioned social cognitive task performance being objectively better than how they expected the average person would perform (DeBrabander et al., 2021). Whether due to accurate or inaccurate self-assessment, autistic adults may perceive themselves as poorer liars than non-autistic adults, contributing to a reduced inclination to lie. Furthermore, moral beliefs about the acceptability of lying may also differ between autistic and non-autistic adults. Viewing lying as morally acceptable is also associated with more frequent (Grant et al., 2019; Hart et al., 2019) and skillful (Wright et al., 2015) deception in non-autistic individuals. However, autistic adults may be more likely to base their judgements of social or moral transgressions upon whether social or moral rules were perceived to be broken (Zalla et al., 2011), and autistic children, adolescents and adults may more often prioritise outcome over intention when making moral judgements (Dempsey et al., 2020). The outcome of a lie (i.e. someone is deceived) may, therefore, be viewed more as more important than intent (e.g. to spare someone’s feelings). Thus, autistic adults may consider deception to be less acceptable than non-autistic adults and, therefore, be less inclined to deceive.

Lying can also be more cognitively demanding than being truthful (Caso et al., 2005; Kireev et al., 2017; Vrij, 2008), relying upon interrelated social and cognitive processing (Sporer, 2016; Talwar & Crossman, 2011). Throughout typical development, non-autistic individuals develop strategies to reduce the cognitive load of lying (see Walczyk & Fargerson, 2019). Faster response times on the Sheffield Lie Test (Spence et al., 2001) – a cognitive test of lie telling – modestly predict (*r* = −0.10) increased frequency of self-reported lying in non-autistic adults (Debey et al., 2015). However, the relationship between cognitive demand and lying in everyday situations may be more pronounced for autistic people. Processing speed is often slower in autistic children, adolescents and adults than non-autistic controls (Velikonja et al., 2019; Zapparrata et al., 2022; also see Ferraro, 2016) and slower processing speed is associated with difficulties in social communication and interaction (Haigh et al., 2018). Lying may, therefore, be a more cognitively demanding task for autistic than non-autistic adults. Indeed, Sip et al. (2008) describe deception as a complex act of social decision-making and autistic adults have difficulty with fast decision-making during social interaction (Luke et al., 2012, see also Ashwin & Brosnan, 2019). As such, the cognitive demand of deception (i.e. slower processing speed) may reduce autistic individuals’ broader inclination to lie to a greater extent than non-autistic adults.

Specific social cognitive and executive function mechanisms associated with lying can differ between autistic and non-autistic adults. These, in turn, may further increase the cognitive difficulty of lying for autistic adults and lead to a reduced inclination to lie. First, it is often argued that the ability to lie is dependent upon ToM (Spence et al., 2004). There is some limited evidence that ToM is positively related to the inclination to lie in non-autistic adults (El Haj et al., 2017). Furthermore, two recent meta-analyses report a robust relationship between ToM and lying in neurotypical children and adolescents (Lee & Imuta, 2021; Sai et al., 2021). It is proposed that ToM difficulties are common (though not universal) for autistic people (Brewer et al., 2017; also see Gernsbacher & Yergeau, 2019; Milton, 2012). However, a relationship between ToM and lying in autistic children and adolescents is only found intermittently (see Bagnall et al., 2022). Two recent studies have proposed that, when lying, autistic individuals may prioritise social cognitive and executive functioning processes other than ToM. Van Tiel et al. (2021) suggested that autistic adults who performed well during a computerised deception task may have used effortful reasoning to learn from observed behaviours rather than ToM. Furthermore, Ma et al. (2019) reported that autistic children’s working memory (though not ToM) was positively associated with lying inclination and ability. Consequently, Ma et al. (2019) proposed that, during deception, autistic children may use working memory to compensate for diminished ToM. Indeed, working memory enables the maintenance of autobiographical and episodic information when non-autistic people lie (Maldonado et al., 2018; Sporer, 2016). However, difficulties with working memory, as well as short- and long-term and episodic memory are common in autism throughout the lifespan (Cooper & Simons, 2019; Crane & Maras, 2018; Desaunay et al., 2020; Habib et al., 2019; Kercood et al., 2014). Difficulty with the retrieval and maintenance of self-related memories may, therefore, further increase the cognitive demand of deception for autistic adults. However, similar to autistic children (see Ma et al., 2019), autistic adults may rely more heavily upon working memory during deception to compensate for difficulties with ToM. In turn, autistic and non-autistic adults with greater working memory capacity may be more inclined to lie in everyday situations.

Understanding autistic adults’ inclination to lie in everyday situations can contribute to knowledge of social vulnerability, as well as informing clinical and forensic practice. First, autistic adults may experience interpersonal difficulties if they are less inclined to lie. For example, autistic people are potentially more susceptible to physical, social and financial exploitation (Barnett & Maticka-Tyndale, 2015; Griffiths et al., 2019) and to becoming involved in criminal activities through manipulation of social naiveite (Bates, 2022; Walter et al., 2021; also see Brewer et al., 2022). Therefore, a lack of inclination and/or ability to lie may hinder autistic adults’ avoidance or extraction from interpersonal victimisation. There are also potential issues for clinical or forensic settings if autistic people are assumed to be inherently truthful (so-called ‘positive stigma’, see Brosnan & Gavin, 2021). For instance, forensic practitioners who encounter autistic people during criminal investigations may assume that they are more likely to be honest, given that truthfulness is a characteristic often attributed to autistic people (Atherton et al., 2019; de Schipper et al., 2016; Huntley et al., 2019). Indeed, many lay persons believe that autistic individuals do not (or cannot) lie (Maras, Marshall, & Sands, 2019). Stereotypes – assumed characteristics and behaviours of specific groups (Hilton & Von Hippel, 1996) – and other cognitive heuristics can influence decision-making in clinical and forensic contexts (Dinos et al., 2015; Featherston et al., 2020; though also see Dehon et al., 2017). Given that victims and/or witnesses sometimes lie to protect themselves or others (Lindholm & Cederborg, 2016; Talwar et al., 2004), a stereotype-driven assumption of honesty from autistic victims, witnesses or suspects may hinder an investigation and potentially also lead investigators to fail to detect signs of such exploitation. In sum, there is a need for a greater understanding of deception in autistic adulthood.

## This study

Lying is a complex form of social communication underpinned by a network of individual and moral beliefs, as well as social cognition and executive functioning processes. Autistic adults’ differences in these areas suggest a potentially reduced inclination to lie in everyday situations compared with non-autistic adults. However, despite receiving some more attention in recent years, deception in autistic adulthood is still not well understood. This study examined (1) whether autistic adults have a reduced inclination to lie in everyday situations compared with non-autistic adults and (2) which factors are associated with autistic and non-autistic adults’ inclination to lie. The following hypotheses were tested:

1. Autistic adults will report an overall reduced inclination to lie in everyday situations compared with non-autistic adults.
2. Self-assessed ability to lie will be positively associated with the inclination to lie in both autistic and non-autistic adults.
3. Viewing lying as morally acceptable with be positively associated with the inclination to lie in both autistic and non-autistic adults.
4. The cognitive demand (i.e. processing speed) of lying will be negatively associated with the inclination to lie in both autistic and non-autistic adults.
5. ToM will be positively associated to the inclination to lie in non-autistic adults, but we tentatively expect there will be no such relationship for autistic adults.
6. Working memory capacity will be positively associated with autistic and non-autistic adults’ inclination to lie in everyday situations.

## Method

### Power analysis

Due to the lack of previous research using self-report deception measures such as the Lying in Everyday Situations Scale (LiES; Hart et al., 2019) with autistic adults, we used response inhibition^1^ to estimate an approximate effect size for group differences in the Sheffield Lie Test (SLT: Spence et al. 2001) scores. Using the (moderate) effect size of *g* = 0.55 (Geurts et al., 2014) for the difference between autistic and non-autistic participants in response inhibition, a priori power analysis using G*Power (Faul et al., 2009) revealed that 34 participants per group were needed to detect group differences in SLT performance at 80% power.

### Participants

Participants were recruited via research volunteer databases and social media. Participants were required to be fluent English speakers, have normal or corrected to normal vision, not have a diagnosed or suspected psychiatric or neurocognitive condition (other than autism) and to be aged between 18 and 60 years.^2^ Seventeen participants were excluded as they failed to meet inclusion criteria (e.g. not using a laptop or desktop computer) or failed checks of attention and understanding (e.g. ‘Please select Option 2’). The final sample comprised of 82 participants (41 autistic and 41 non-autistic). Twenty-nine of the autistic group identified as female, 10 as male, one as non-binary and one preferred not to say. All autistic participants stated that they had received a clinical diagnosis of an autism spectrum condition. Twenty-eight of the non-autistic group identified as female (and 13 as male). As expected, the autistic group (*M* *=* 39.41) had significantly higher autism quotient scores (AQ-50: Baron-Cohen et al., 2001) than the non-autistic group (*M* *=* 14.41).^3^ The non-autistic group were significantly younger (*M* *=* 25.31 years) than the autistic group (*M* *=* 32.46). Educational level – measured by the International Standard Classification in Education (UNESCO, 2012)^4^ – was used as a proxy for intelligence quotient (IQ) in line with other online autism research (Livingston et al., 2020; though also see Keen et al., 2016), with no significant difference in education level between the autistic and non-autistic groups (See Table 1).

**Table 1.** Mean scores, standard deviations and *t*-test results for age, AQ-50 and education level for autistic and non-autistic groups.

	Autistic adults (*n* *=* 41)			Non-autistic adults (*n* *=* 41)			*t*	*p*	*d*	95% CI
	*M*	SD	Range	*M*	*SD*	Range				
Age	32.46	10.51	19–56	25.31	7.50	18–45	3.54	< 0.001	0.782	(0.330, 1.22)
AQ-50	39.41	6.71	17–48	14.41	6.67	2–36	16.91	< 0.001	3.73	(3.00, 4.45)
Education level	5.80	1.38	3–8	6.26	1.04	4–8	−1.71	0.091	−0.378	(0.813, 0.060)

Educational level (International Standard Classification in Education – UNESCO, 2012).

AQ-50: Autism Quotient-50; CI: confidence interval.

### Measures

The LiES Scale (Hart et al., 2019) is a 14-item questionnaire that measures an individual’s inclination to lie in everyday life (e.g. ‘I lie to stay out of arguments with people’) on a seven-point Likert-type scale from strongly disagree (1) to strongly agree (7). Obtainable scores range between 14 and 98, with higher scores representing a greater inclination to lie. LiES scores correlate positively with frequency of lies told during the past 24 h (Hart et al., 2019). The LiES is reported to be a valid and reliable measure of deception inclination (Hart et al., 2019), demonstrating construct validity with other deception measures (e.g. the Cole Partner Deception Scale; Cole, 2001) and convergent validity with related constructs such as ‘bullshitting’ (Littrell et al., 2021).

The Revised Lie Acceptability Scale (RLAS; Oliveira & Levine, 2008) measures attitudes about the acceptability of lying, containing eight statements (e.g. ‘Lying is immoral’) on a seven-point Likert-type scale (1 = strongly disagree; 7 = strongly agree). The average of all responses is used as the total score, with obtainable total scores ranging from 1 to 7 (with higher scores indicating higher levels of lie acceptability). The RLAS is reported to be a valid measure of attitudes about deception behaviour (Oliveira & Levine, 2008), and is commonly used with deception-related measures (Grant et al., 2019; Hart et al., 2019; Littrell et al., 2021).

SLT (Spence et al., 2001) is a computer-based cognitive test of lying which measures reaction time (RT) in milliseconds on trials when participants tell the truth and when they lie. The SLT measures the cognitive difficulty of lying based upon the assumption that lying elicits longer RTs than truth-telling. In the meta-analysis by Suchotzki et al. (2017), SLT lie trials were on average 180 milliseconds longer than truth trials (the ‘lie effect’). The SLT elicited the largest RT difference between truth and lie trials of all computer-based deception tasks included (Suchotzki et al., 2017). In the pre-test stage of the SLT, participants submit yes/no responses to a set of 40 questions (see Supplemental Appendix 1) that relate to autobiographical events (e.g. ‘Have you made a phone call in the past 24 hours?’). Pre-test responses provide a baseline for participants’ subsequent truthful and deceptive test responses. During the test, each of the 40 questions is presented twice: once when participants are cued to answer honestly (‘Truth’ appears above the question) and once when prompted to answer dishonestly (‘Lie’ appears above the question). Participants are instructed to answer as quickly and as accurately as possible by pressing the left (‘4’) or right (‘6’) key for YES or NO. The response labels ‘YES’ and ‘NO’ are presented beneath each question on the left or right (counterbalanced across participants). Participants’ RTs on truth trials (SLT truth speed) and lie trials (SLT lie speed) are produced, as is lie effect score (SLT lie effect), calculated through deducting the truth trials RT from lie trials RT (as per Debey et al., 2015; Suchotzki et al., 2017).

The Automated Operation Span Task (AOSPAN; Turner & Engle, 1989; Unsworth et al., 2005) is a computer-based test of verbal working memory capacity. During each trial, participants are presented with a distraction maths question. At the end of each trial, a letter is presented to the participant (e.g. L). At the end of each set of trials, participants are presented with a 3 × 4 grid of letters. From this grid, participants are asked to select the letters presented previously in the correct order. The total AOSPAN score is the total number of letters recalled in the correct order during test trials (maximum achievable score = 75). The AOSPAN has good internal consistency (alpha = 0.78) and test–retest reliability (0.83; Unsworth et al., 2005.)

The Frith-Happé Animations Test (Abell et al., 2000; Livingston et al., 2021; White et al., 2011) is a measure of ToM. Participants watch 12 animations during which two triangles interact with one another. As per White et al. (2011) and Livingston et al. (2021), following each animation, participants are asked to choose which of three categories (no interaction, physical interaction or mental interaction) best describes the interaction. A maximum total score of 12 can be achieved across the three domains (with a maximum score of 4 within each domain). Scores for each domain are converted into percentage accuracy. The Frith-Happé Animations Test is valid, reliable and has been used in online formats with autistic participants (see Livingston et al., 2021).

### Procedure

The study was presented in an online Qualtrics survey in a quasi-randomised format. Participants completed the study remotely using a desktop or laptop computer. After providing demographic information, participants were provided with a working definition of deception (see Supplemental Appendix 2). Participants then completed LiES, RLAS, a self-rated assessment of lying ability on a 10-point Likert-type scale (1 = very poor; 10 = excellent) and the Frith-Happé animations task in Qualtrics. The SLT and AOSPAN task were completed through Inquisit 5. Upon completion, participants were fully debriefed. Participants were reimbursed for their time with a £13 shopping voucher. The study received ethical approval from the Psychology Research Ethics Committee at the University of Bath.

### Statistical analysis

All analyses were performed using SPSS (version 28). One non-autistic participant was removed from the data set due to missing SLT scores. Two non-autistic participants were substantially older (> 3 SDs) and these outliers were removed from the data set. These removals resulted in the final sample of 41 autistic and 41 non-autistic participants. As the autistic group were significantly older than the non-autistic group, age was controlled for as a covariate during analyses. Heterogeneity of variance was observed in LiES, SLT truth and SLT lie speed^5^ scores (Levene’s test *p* < 0.05). A series of transformations failed to improve normality of distribution in residual scores for self-rated lie ability (based on 10-point Likert-type scale question) and non-normal distribution in Frith Happé residual scores. Bootstrapped Bias corrected accelerated (BCa) 95% confidence intervals (CIs) for estimated group means and/or effect sizes were produced for all analyses to account for these violations (Field & Wilcox, 2017). To correct for multiple comparisons, the false discovery rate (FDR) method was applied (Benjamini & Hochberg, 1995) for the analysis of covariance (ANCOVA) and Pearson’s partial correlation analyses.

### Community involvement statement

Members of the autism community were not involved in the design, implementation or interpretation of this study.

## Results

### Autistic and non-autistic group comparisons

We first performed a series of one-way ANCOVAs (controlling for age) to compare group scores for the inclination to lie in everyday situations and associated measures (see Table 2). There was no significant difference in the inclination to lie (LiES scores) between autistic and non-autistic participants, nor levels of lie acceptability (RLAS scores). Autistic and non-autistic participants did not significantly differ on SLT truth speed or SLT lie effect scores. Autistic participants’ SLT lie speed was initially significantly slower than in the non-autistic group, although this group difference was no longer significant following the FDR correction for multiple comparisons. Self-rated ability to lie scores were significantly higher in non-autistic participants than non-autistic participants. Finally, there were no significant differences between autistic and non-autistic participants in ToM (Frith-Happé animation scores) or working memory (AOSPAN scores).

**Table 2.** Task performance in autistic and non-autistic adults using estimated (*M*_adj_) mean scores (controlling for age).

	Autistic adults (*n* *=* 41)			Non-autistic adults (*n* *=* 41)			*f*	*p*	Adjusted *p*	Partial η^2^
	*M*_adj_	SE	BCa 95% CI	*M*_adj_	SE	BCa 95% CI				
LiES	35.28	1.67	31.85, 38.41	38.40	1.24	35.92, 40.75	1.99	0.162	0.259	0.025
RLAS	3.12	0.17	2.81, 3.45	3.34	0.12	3.12, 3.55	1.05	0.308	0.319	0.013
SLT truth speed	2357.57	90.83	2193.29, 2519.41	2142.69	64.50	2020.63, 2279.12	3.47	0.066	0.132	0.042
SLT lie speed	2601.29	95.13	2417.40, 2792.66	2324.23	60.88	2201.03, 2442.96	5.58	0.021	0.084	0.066
SLT lie effect	−243.69	38.04	−319.40, −172.32	−181.55	32.53	−246.10, −115.78	1.18	0.280	0.319	0.015
Self-rated lie ability	4.56	0.39	3.74, 5.33	6.52	0.38	5.77, 7.29	12.54	0.001	**0.008**	0.137
Frith-Happé (%)	65.65	4.36	56.58, 74.25	72.76	4.83	62.54, 81.47	1.01	0.319	0.319	0.013
AOSPAN	38.79	3.06	32.79, 44.31	47.63	2.79	42.35, 53.41	4.07	0.047	0.125	0.049

BCa: bias corrected accelerated 95% standard errors (SEs) and confidence intervals (CIs) for *M*_adj_; LiES: lying in everyday situations; RLAS: Revised Lie Acceptability Scale; SLT: Sheffield Lie Test; Frith Happé (%): Theory of Mind animated triangles task; AOSPAN: Automated Operation Span Task.

Adjusted *p* = FDR correction. Bold values indicate significance after FDR correction.

### Correlation analyses

We then conducted two separate Pearson’s Partial Correlation analyses (controlling for age) to examine the relationships between the previously reported variables and LiES scores in autistic and non-autistic groups (see Table 3).^6^ There was a significant relationship between LiES and RLAS scores for both autistic and non-autistic participants. In the autistic group, SLT truth speed, SLT lie speed and self-rated ability to lie all significantly correlated with LiES scores. SLT lie effect was initially significantly correlated with LiES scores in the autistic group, although following FDR correction this relationship was of marginal significance and BCa 95% CIs for *r* overlapped with zero, therefore, indicating a non-significant relationship (Hayes, 2009). In the non-autistic group, Frith Happé animations and AOSPAN scores significantly correlated with LiES scores. All other partial correlations were non-significant.

**Table 3.** Partial Pearson correlations for LiES scores (controlling for age).

	*r*	BCa 95% CI for *r*		*p*	Adjusted *p*
		Lower	Upper		
Autistic group					
RLAS	0.65	0.47	0.80	0.001	**0.002**
SLT truth speed	−0.38	−0.61	−0.10	0.016	**0.028**
SLT lie speed	−0.52	−0.69	−0.27	0.001	**0.002**
SLT lie effect	0.32	−0.04	0.59	0.042	0.058
Self-rated lie ability	0.52	0.26	0.71	0.001	**0.002**
Frith-Happé	0.02	−0.33	0.35	0.929	0.929
AOSPAN	0.14	−0.16	0.43	0.383	0.446
Non-autistic group					
RLAS	0.41	0.15	0.63	0.008	**0.028**
SLT truth speed	0.07	−0.29	0.42	0.671	0.819
SLT lie speed	0.03	−0.31	0.35	0.846	0.846
SLT lie effect	0.06	−0.27	0.41	0.702	0.819
Self-rated lie ability	0.22	−0.05	0.45	0.165	0.288
Frith-Happé	−0.53	−0.72	−0.35	0.001	**0.007**
AOSPAN	−0.39	−0.64	−0.05	0.014	**0.032**

LiES: lying in everyday situations; BCa: bias corrected accelerated 95% confidence intervals (CIs) for *r*; RLAS: Revised Lie Acceptability Scale; SLT: Sheffield Lie Test; Frith Happé: Theory of Mind animated triangles task; AOSPAN: Automated Operation Span Task.

Adjusted *p* *=* FDR correction. Bold values indicate significance after FDR correction.

### Regression analyses

Finally, we carried out two hierarchical multiple regression analyses (one for the autistic group, the second for the non-autistic group) to examine the degree to which significant correlations explained variation in LiES scores.

In the autistic group (see Table 4),^7^ age alone (Step 1) did not make a significant contribution to LiES scores, *p* = 0.331. RLAS scores (Step 2) explained an additional 41.3% of the variance, significantly improving the model, *p* < 0.001. The addition of SLT lie speed (Step 3) explained a further 9.8% of variance in LiES scores, *p* = 0.008. Self-rated lie ability scores (Step 4) explained 6.1% of the variance, *p* = 0.023. The final model explained 59.5% of the variance in LiES scores, *F*(4, 36) = 13.25, *p* < 0.001, adjusted *R*^2^ = 0.551.

**Table 4.** Hierarchical multiple regression predicting LiES scores in autistic participants.

	Model 1			Model 2			Model 3			Model 4		
	*B*	BCa95% CI	β	*B*	BCa95% CI	β	*B*	BCa 95% CI	β	*B*	BCa95% CI	β
Age	−0.16	−0.49, 0.12	−0.16	0.03	−0.25, 0.32	0.03	0.07	−0.18, 0.31	0.07	0.09	−0.14, 0.29	0.08
RLAS				6.91***	4.15, 10.69	0.67	5.73***	3.06, 9.50	0.55	4.81***	2.65, 7.97	0.47
SLT lie speed							−0.006**	−0.011, −0.002	−0.34	−0.005*	−0.009, −0.002	−0.29
Self-rated lie ability										1.22*	0.16, 2.33	0.27
*R* ^2^	0.02			0.44			0.54			0.60		
*F*	0.97			14.74***			14.19***			13.25***		
*∆R* ^2^	0.02			0.41			0.10			0.06		
*∆F* ^2^	0.97			27.83***			7.81**			5.39*		

LiES: lying in everyday situations; BCa: bias corrected accelerated; CI: confidence interval; RLAS: Revised Lie Acceptability Scale; SLT: Sheffield Lie Test; *∆*: change.

* *p* < 0.05, **p < 0.01, ***p < 0.001 = unadjusted *p* values.

In the non-autistic group (see Table 5), age (Step 1) did not significantly explain variation in LiES scores, *p* = 0.139. The addition of RLAS scores (Step 2) made a significant contribution to the model, explaining 16.2% of the variance, *p* = 0.008. Frith-Happé animations scores (Step 3) explained 17.6% of the variance in LiES scores, *p* = 0.002. AOSPAN scores (Step 4) contributed 6.2% to the variance at marginal significance, *p* = 0.051. The final model explained 45.5% of the variance in LiES scores, *F*(4, 36) = 7.50, *p* < 0.001, adjusted *R*^2^ = 0.394.

**Table 5.** Hierarchical multiple regression predicting LiES scores in non-autistic participants.

	Model 1			Model 2			Model 3			Model 4		
	*B*	BCa 95% CI	β	*B*	BCa95% CI	β	*B*	BCa 95% CI	β	*B*	BCa 95% CI	β
Age	−0.24	−0.62, 0.15	−0.24	−0.19	−0.48, 0.14	−0.19	−0.24	−0.49, 0.06	−0.23	−0.29*	−0.53, −0.02	−0.28
RLAS				4.27*	1.17, 7.15	0.40	2.97*	0.05, 5.71	0.28	3.13*	0.17, 5.67	0.29
Frith-Happé							−0.11*	−0.18, −0.05	−0.44	−0.09*	−0.16, −0.02	−0.35
AOSPAN										−0.12	−0.23, −0.001	−0.27
*R* ^2^	0.05			0.22			0.39			0.46		
*F*	2.29			5.26*			7.98***			7.50***		
*∆R* ^2^	0.05			0.16			0.18			0.06		
*∆F* ^2^	2.29			7.84**			10.71**			4.08*		

LiES: lying in everyday situations; BCA: bias corrected accelerated; CI: confidence interval; RLAS: Revised Lie Acceptability Scale; Frith Happé animations: Theory of Mind triangles task; AOSPAN: Automated Operation Span Task; *∆*: change.

* *p* < 0.05, **p < 0.01, ***p < 0.001 = Unadjusted *p* values.

## Discussion

The difficulty that autistic children and adolescents can experience with lying (Bagnall et al., 2022) and the increasing socio-complexity of deception post-childhood (Walczyk & Fargerson, 2019) would suggest a reduced inclination to lie in autistic adulthood. However, we found that autistic and non-autistic adults did not significantly differ in their reported inclination to lie in everyday situations (based upon LiES scores). While autistic adults did report lower LiES scores than non-autistic adults, with a small effect size in the hypothesised direction, this difference was not statistically significant. However, this finding may be seen as consistent with autistic adults’ inclination to engage in computerised deception tasks (van Tiel et al., 2021) and similar verbal deception to non-autistic adults in mock-police suspect interview settings (Bagnall et al., 2023). That autistic and non-autistic adults did not significantly differ in their inclination to lie in everyday situations also overlaps with certain adaptive social behaviours in autism. For instance, camouflaging – a social strategy to disguise autism characteristics (Cage & Troxell-Whitman, 2019) – demonstrates similar inclination for identity management and self-protection. Indeed, the experience of camouflaging has been described by some autistic adults as akin to deception (Hull et al., 2017), as has the related construct of compensation (Livingston et al., 2019b). Autobiographical narratives from some autistic adults also describe the withholding and reconstruction of personal information as a self-protection strategy (Davidson & Henderson, 2010). However, this also highlights the potential for deception to have personal and/or legal consequences for autistic adults in forensic contexts. Autistic people are often more susceptible to compliance (Chandler et al., 2019) and as such may be manipulated into criminal activity (North et al., 2008; also see Brewer at al., 2022) and may, therefore, lie or conceal information to protect others. As discussed, investigators may be more likely to assume that autistic people do not lie, and reliance on stereotypes can lead to ‘tunnel vision’ (i.e. confirmation bias which excludes contradictory information) during investigative interviewing (Minhas et al., 2017). Indeed, Maras, Crane, et al. (2019) found that autistic mock-witnesses are rated as more credible than non-autistic mock-witnesses when their diagnosis is disclosed to raters, despite no difference in their testimony. Future research should examine the motivators for deception in autistic adults (e.g. protection of self or others, etc.) and how this deception is displayed.

Both autistic and non-autistic adults who viewed lying as more morally acceptable were more inclined to deceive in everyday situations. These findings, therefore, extend previous research showing this relationship in neurotypical samples (Hart et al., 2019; Littrell et al., 2021). It is of note that autistic and non-autistic adults did not significantly differ in their perception of the acceptability of lying. However, much of our understanding of moral reasoning and social behaviour in autism is based upon child and adolescent samples (Bellesi et al., 2018). Bellesi et al. (2018) reported similar rates between autistic and non-autistic adults in frequency and perceived acceptability of social transgressions in everyday scenarios (for a review of morality in autism, see Dempsey et al., 2020).

Self-rated ability to lie predicted autistic adults’ broader inclination to lie in everyday situations. Autistic adults in this study also rated themselves as significantly poorer liars compared to the non-autistic group. These findings may provide support to the proposition that autistic people find LiES to be more socially and cognitively demanding than non-autistic people. However, experiences of stigma may contribute to autistic people internalising stereotypical beliefs about autism (Han et al., 2022). As such, self-report scores relating to the ability to lie may be influenced by broader societal perceptions of autism and deception. Indeed, like non-autistic people, autistic individuals’ beliefs are susceptible to the effects of social conformity (Lazzaro et al., 2019). How autistic people perceive neurotypical conceptions of autism and honesty is a direction for future research. However, our findings emphasise the value in not only investigating social behaviour in autism through empirical measures of social cognition, but also by examining how attitudes and beliefs influence behaviour (Atherton et al., 2019; Oliveira & Levine, 2008).

RT on SLT lie trials was found to be a significant predictor of the inclination to lie for autistic adults. However, this relationship was not found for non-autistic adults (more on this point later). Specifically, autistic adults who lied more slowly were also less inclined to lie in everyday situations, and those who lied more quickly were more inclined to lie. There are two potential interpretations for this finding. First, the cognitive demand of lying influences autistic adults’ inclination to lie. Second, general processing speed influences autistic adults’ inclination to lie. In support of this second interpretation, autistic participants’ RTs were (non-significantly) slower than non-autistic controls on SLT truth trials (small–moderate effect size) and SLT lie trials (moderate effect size). As such, the significant relationship between SLT lie speed and the inclination to lie may reflect generally slower processing speed. As discussed, processing speed is typically slower in autistic populations (Zapparrata et al., 2022) and slower processing speed is related to a range of psychosocial behaviours (Duncan et al., 2019; Haigh et al., 2018; Pallathra et al., 2019). However, the larger effect size associated with SLT lie speed may support the interpretation that it is the cognitive demand of lying which influences autistic adults’ inclination to lie. Furthermore, the correlation of SLT lie speed and LiES scores was stronger than SLT truth speed and LiES scores in the autistic group. In addition, while the SLT lie effect (the difference in RT between truth and lie trials) for non-autistic participants in this study is consistent with that reported in the meta-analysis of Suchotzki et al. (2017) (180 milliseconds), autistic adults’ SLT lie effect was (non-significantly) larger with a small effect size. Whether autistic adults experience greater cognitive demand when lying (and how this relates to broader social behaviour) is a direction for future research.

Neither self-reported ability to lie nor SLT performance was associated with non-autistic adults’ inclination to lie. On the surface, this appears contrary to previous research. However, in previous studies, self-reported deception ability (Verigin et al., 2019) and SLT lie effect (Debey et al., 2015) were related to frequency of lies told in the previous 24 h. Whereas our measure, the LiES (Hart et al., 2019), assesses a general tendency to lie across different everyday situations. Although LiES scores positively correlate with the number of lies told during the past 24 h (Hart et al., 2019), the LiES does not directly measure lie-telling frequency. The LiES may primarily measure inclination to engage in deception, which is distinct but related to how often one actually lies. Similar to deception ability (Walczyk & Fargerson, 2019), a general inclination to lie in everyday situations may be a normative trait produced through typical development (Talwar & Crossman, 2022). Indeed, non-autistic adults’ general cognitive ability is not associated with their LiES scores (Littrell et al., 2021). Therefore, socio-cognitive demand incurred through lying may be less influential for non-autistic adults’ inclination to lie than moral and social factors (see below).

Contrary to expectation, non-autistic adults with greater ToM and working memory capacity were less inclined to deceive others. In line with the previous discussion, these findings may point to a surprising though intuitive answer. Non-autistic individuals who have greater awareness of others’ mental states (ToM) and greater capacity to maintain context-relevant information (working memory) may be less inclined to lie due to, for instance, recognising the potential harm that deception may cause for themselves and others. Indeed, ToM and working memory are each positively associated with pro-social behaviour in neurotypical childhood (de Wilde et al., 2016; Imuta et al., 2016). Furthermore, ToM ability corresponds with non-autistic adolescents concealing less information and being more forthcoming in close relationships (Lavoie & Talwar, 2022). As such, greater ToM capacity may facilitate honest interactions and reduce the need for deception (for further discussion, see Lavoie & Talwar, 2022). Indeed, it appears that only one previous study has reported an association between ToM and deception inclination in non-autistic adults (El Haj et al., 2017). In that study, a subset of five questions from the Impression Management (IM) subscale of the Paulhus Deception Scale (Paulhus, 1998) were used to measure deception inclination, with the abbreviated IM subscale positively correlating with cognitive ToM (El Haj et al., 2017). However, IM is not synonymous with lying to others (Uziel, 2014), and the five questions extracted from the full IM subscale do not all specifically address deceptive behaviour (e.g. ‘There have been occasions when I have taken advantage of someone’). As such, ToM and working memory may only be positively related to specific phases of neurotypical deception, such as during the development of lying ability (Sai et al., 2021) or actively engaging in deceptive behaviour (Walczyk et al., 2014).

It is possible that effortful, compensatory strategies may help explain the lack of a relationship between ToM and working memory with autistic adults’ inclination to lie. Compensatory strategies draw more heavily upon cognitive ability to, for example, infer others’ perspectives or engage in neurotypical social behaviour through deliberative reasoning (Ashwin & Brosnan, 2019; Livingston, Colvert, et al., 2019). Deliberative reasoning is reflected in greater processing time when compared to rapid intuitive processing within Dual Process Theories of cognition (see Evans & Stanovich, 2013). The moderately sized (albeit non-significant) effect of autistic adults’ trend towards slower SLT lie speeds may reflect enhanced deliberative processing relative to intuitive processing. This would be consistent with the Dual Process Theory of Autism (Ashwin & Brosnan, 2019; Brosnan & Ashwin, 2022, 2023; Brosnan et al., 2017, 2016); Such non-neurotypical strategies have been suggested to underpin deception inclination and ability in autistic children (Ma et al., 2019) and adults (van Tiel et al., 2021). The higher cognitive cost of deliberative strategies may mean that some autistic individuals are less inclined to engage in such strategies and therefore appear less socially motivated (Livingston et al., 2019a). Indeed, findings in this study indicate that autistic adults for whom deception is less cognitively demanding (or have faster general processing speed) are also more inclined to lie in everyday situations. However, the potential alternative mechanisms underpinning these abilities are beyond the scope of this study and require further investigation.

There are several limitations to this study. Despite instructing participants to complete the study in a quiet environment free from distractions, we had little control over participants’ online testing conditions. A further limitation is that we did not request evidence of a formal autism diagnosis. However, the majority of autistic participants were recruited while the study was only advertised to an autism research participant database. The robust AQ-50 group differences obtained in the study further indicate that autistic participants were consistent with the necessary clinical criteria. A further substantial limitation is that we only used one measure for each construct under investigation. For example, an inclination to lie as measured by LiES scores (Hart et al., 2019). Despite this, our lie inclination findings fit within an emerging body of research relating to autistic adults’ ability to manage others’ expectations by selectively choosing when and how to disclose information (Cage & Troxell-Whitman, 2019; Hull et al., 2017; Livingston, Colvert, et al., 2019), as well as engage successfully in computerised deception tasks (van Tiel et al., 2021) and tell verbal lies when instructed (Bagnall et al., 2023). However, future research should use multiple measures to investigate autistic peoples’ inclination to lie in everyday situations. Further to this point, we only used a single measure for ToM (Frith-Happé animations) and working memory (AOSPAN). Unlike Livingston et al. (2021), we found no significant difference in Frith-Happé animations scores between autistic and non-autistic participants. As explained, non-autistic adults’ scores in this study were non-normally distributed (strong negative skew), with bootstrapping applied to correct for this violation (Field & Wilcox, 2017). It should also be noted that, in our study, both the autistic group and the non-autistic group scored substantially higher on the Frith-Happé animations test than in the study by Livingston et al. (2021). Given mixed findings on a relationship between ToM and deception in autistic children and adolescents (Bagnall et al., 2022; Ma et al., 2019), future research should also include other ToM measures (e.g. Adult-Theory of Mind; Brewer et al., 2017) to examine whether mentalising ability is related to autistic adults’ inclination or ability to lie. In sum, given the use of single measures, the lack of relationship between ToM and working memory with autistic adults’ inclination to lie in this study should therefore be interpreted with caution.

The potential impact of the participant sample in this study should also be considered. Our sample was comprised of more females than males in the autistic group (29/41) and the non-autistic group (28/41). As it is currently estimated that the male to female gender ratio in autism is approximately 3:1 (Loomes et al., 2017), our participant sample is, therefore, not representative of the general autistic population. This overrepresentation of autistic female participants is consistent with sampling in online autism research more broadly (Rødgaard et al., 2022). Autistic females are reported to have higher processing speed and executive function compared with autistic males (Lehnhardt et al., 2016), which would potentially aid an ability to lie in everyday situations. Furthermore, autistic females are more likely to engage in camouflaging (Hull et al., 2020; McQuaid et al., 2022; Wood-Downie et al., 2021), which as discussed shares certain similarities with deception (e.g. impression management). Indeed, studies with evenly matched proportions of female and male autistic participants have reported non-significant autistic and non-autistic group differences in reputation management (Gernsbacher et al., 2020). The aforementioned van Tiel et al. (2021) study, in which autistic participants demonstrated intact inclination and ability to deceive a computerised opponent had a similarly even male/female gender split. Future research should examine deception behaviours in sufficiently large autistic samples to detect meaningful gender differences or recruit single-gender participant samples so as to determine the generalisability of emerging findings of deception in autistic adulthood.

## Conclusion

This study makes two contributions to autism and deception literature. First, by challenging stereotypical assumptions about autism by demonstrating that autistic adults are not necessarily less inclined to lie in everyday situations than non-autistic adults. This highlights the need to better understand the contexts in which autistic adults lie, as well as for clinical and forensic practitioners to be conscious of how stereotype-driven perceptions of autism may impact their decision-making. Second, this study highlights potential factors which underpin a general inclination to lie in autistic and non-autistic populations. Consistent with previous studies, the degree to which (autistic and non-autistic) participants perceived lying as morally acceptable was positively associated with how inclined they were to lie in everyday situations. The remaining factors differed between autistic and non-autistic groups. For autistic adults, poorer self-assessed lying ability and slower lie speed were associated with a reduced inclination to lie. For non-autistic adults, a reduced inclination to lie was associated with increased ToM and working memory capacity. Taken together, these findings reflect the importance of investigating social behaviour in autism in relation to beliefs and attitudes as well as (social) cognition.

## Supplemental Material

sj-docx-3-aut-10.1177_13623613231183911 – Supplemental material for Autistic adults’ inclination to lie in everyday situations Supplemental material, sj-docx-3-aut-10.1177_13623613231183911 for Autistic adults’ inclination to lie in everyday situations by Ralph Bagnall, Ailsa Russell, Mark Brosnan and Katie Maras in Autism

sj-rtf-1-aut-10.1177_13623613231183911 – Supplemental material for Autistic adults’ inclination to lie in everyday situations Supplemental material, sj-rtf-1-aut-10.1177_13623613231183911 for Autistic adults’ inclination to lie in everyday situations by Ralph Bagnall, Ailsa Russell, Mark Brosnan and Katie Maras in Autism

sj-rtf-2-aut-10.1177_13623613231183911 – Supplemental material for Autistic adults’ inclination to lie in everyday situations Supplemental material, sj-rtf-2-aut-10.1177_13623613231183911 for Autistic adults’ inclination to lie in everyday situations by Ralph Bagnall, Ailsa Russell, Mark Brosnan and Katie Maras in Autism

## Notes

1. The SLT (and deception more broadly) involves inhibition of truthful responses (Debey et al., 2015; Spence et al., 2004; Suchotzki et al., 2017).

2. Lying speed and frequency declines throughout adulthood, with those aged 60+ being least skilled and least frequent liars (Debey et al., 2015).

3. One non-autistic participant scored 36 on the AQ-50 and one autistic participant scored 17. Although 26 is considered a lower level of clinical cut off for autism (Woodbury-Smith et al., 2005), these two participants were retained in the data set. Resulting group means (see Table 1) were clearly above and below the aforementioned clinical cut-off. The pattern of the results remained the same with and without these two participants included in the analysis.

4. Primary school = 1; Key Stage 3 (secondary school, pre-GCSE) = 2; GCSEs or equivalent = 3; A-levels or vocational equivalent = 4; Short-Cycle Higher Education (Foundation degrees/Higher National Diplomas/Diplomas of Higher Education or equivalent level) = 5; Bachelor’s degree (or equivalent level) = 6; Master’s degree (or equivalent level) = 7; Doctoral (or equivalent level) = 8.

5. A log-10 transformation successfully produced homogeneity of variance in SLT lie speed scores. As the same pattern of results were retained when ANCOVAs were performed on transformed and untransformed SLT lie speed scores, the original untransformed results are presented for ease of interpretation.

6. Supplemental Appendix 3 provides full correlation matrix between all measured variables.

7. SLT truth speed was excluded from the regression model due to being highly correlated with SLT lie speed (*r* = 0.91).
