# Supplementary material for: Autistic adults’ inclination to lie in everyday situations
Source: Autism. 2023 Aug 12;28(3):718–31. doi: 10.1177/13623613231183911 (PMC10913365; doi:10.1177/13623613231183911)
Supplement: sj-docx-3-aut-10.1177_13623613231183911 – Supplemental material for Autistic adults’ inclination to lie in everyday situations [file sj-docx-3-aut-10.1177_13623613231183911.docx]

**Appendix 3. Full correlation matrix**

**Table 6.** Partial Pearson Correlation Matrix (controlling for age) in autistic (*n =* 41) and non-autistic (*n =* 41) groups.

|  | LiES | RLAS | Truth speed | Lie speed | Lie effect | Lie ability | Frith-Happé | AOSPAN |
| --- | --- | --- | --- | --- | --- | --- | --- | --- |
| LiES | - | .65*** | -.38* | -.52*** | .32 | .52*** | .02 | .14 |
| RLAS | .41** | - | -.28 | -.33* | .14 | .37* | .01 | .07 |
| SLT truth speed | .07 | .11 | - | .90*** | .18 | -.25 | -.15 | .08 |
| SLT lie speed | .03 | .05 | .83*** | - | -.26 | -.28 | -.22 | .09 |
| SLT lie effect | .06 | .10 | .28 | -.32* | - | .09 | .16 | -.03 |
| Self-rated lie ability | .22 | .32* | -.07 | -.05 | .03 | - | -.07 | .16 |
| Frith-Happé | -.53*** | -.28 | -.17 | -.02 | -.24 | .04 | - | .16 |
| AOSPAN | -.39* | -.03 | -.09 | -.07 | -.04 | .19 | .30 | - |

Note: Autistic group correlations are presented above the dividing line, non-autistic group correlations are below the line.

LiES: Lying in Everyday Situations; RLAS: Revised Lie Acceptability Scale; SLT: Sheffield Lie Test; Frith Happé animations: Theory of Mind triangles task; AOPSAN: Automated Operation Span Task.

*p < 0.05, **p < 0.01, ***p < 0.001. = Unadjusted *p* values
