# Supplementary material for: Autistic adults’ inclination to lie in everyday situations
Source: Autism. 2023 Aug 12;28(3):718–31. doi: 10.1177/13623613231183911 (PMC10913365; doi:10.1177/13623613231183911)
Supplement: sj-rtf-1-aut-10.1177_13623613231183911 – Supplemental material for Autistic adults’ inclination to lie in everyday situations [file sj-rtf-1-aut-10.1177_13623613231183911.rtf]

Appendix 1. Questions presented to participants to establish 'baseline truth' for lie vs. truth responses during Sheffield Lie Test task

Anticipated yes questions (n=20)	Anticipated no questions (n=20)	
Have you opened a bin in the last 24 hours?	Have you been for a run in the last 24 hours?	
Have you closed a door in the last 24 hours?	Have you eaten chocolate cake in the last 24 hours?	
Have you had a shower in the last 24 hours?	Have you had a bath in the last 24 hours?	
Have you used a knife in the last 24 hours?	Have you made a sandwich in the last 24 hours?	
Have you used a phone in the last 24 hours?	Have you written a letter in the last 24 hours?	
Have you used the internet in the last 24 hours?	Have you lit a candle in the last 24 hours?	
Have you made the bed in the last 24 hours?	Have you read a magazine in the last 24 hours?	
Have you washed your hands in the last 24 hours?	Have you spoken to a police officer in the last 24 hours?	
Have you watched television in the last 24 hours?	Have you broken a glass in the last 24 hours?	
Have you drunk water in the last 24 hours?	Have you drunk fruit juice in the last 24 hours?	
Have you done the washing up in the last 24 hours?	Have you listened to the radio in the last 24 hours?	
Have you got out of bed in the last 24 hours?	Have you signed a document in the last 24 hours?	
Have you sent an email in the last 24 hours?	Have you drunk coffee in the last 24 hours?	
Have you opened a fridge in the last 24 hours?	Have you eaten onions in the last 24 hours?	
Have you used a computer in the last 24 hours?	Have you cleaned a window in the last 24 hours?	
Have you turned on a tap in the last 24 hours?	Have you stroked a pet in the last 24 hours?	
Have you lifted a toilet seat in the last 24 hours?	Have you smoked a cigarette in the last 24 hours?	
Have you listened to music in the last 24 hours?	Have you looked at a watch in the last 24 hours?	
Have you boiled water in the last 24 hours?	Have you vacuumed a carpet in the last 24 hours?	
Have you walked down some stairs in the last 24 hours?	Have you watered some plants in the last 24 hours?	
