# Supplementary material for: Autistic adults’ inclination to lie in everyday situations
Source: Autism. 2023 Aug 12;28(3):718–31. doi: 10.1177/13623613231183911 (PMC10913365; doi:10.1177/13623613231183911)
Supplement: sj-rtf-2-aut-10.1177_13623613231183911 – Supplemental material for Autistic adults’ inclination to lie in everyday situations [file sj-rtf-2-aut-10.1177_13623613231183911.rtf]

Appendix 2. Definition of lying presented to participants.

Please read the following definitions carefully and keep them in mind while answering the survey questions:  
 
- To lie is to make a false statement to another person, with the intention of making that person believe the statement is true. As such, to lie to try and create a false belief in another person. 

- Misremembering (and giving false information without meaning to) is not the same as lying.

- A successful lie means that the intended false-belief has been produced.

- An unsuccessful lie means that the intended false-belief has not been produced.
